# TSA-PACT: a method for tissue clearing and immunofluorescence staining on zebrafish brain with improved sensitivity, specificity and stability

**DOI:** 10.1186/s13578-023-01043-1

**Published:** 2023-05-26

**Authors:** Kang Wang, Yuxin Yu, Yinhui Xu, Yingzi Yue, Fang Zhao, Wenyang Feng, Yijie Duan, Weicheng Duan, Jingjing Yue, Zhiyun Liao, Peng Fei, Hui Sun, Bo Xiong

**Affiliations:** 1 Department of Forensic Medicine, Tongji Medical College, Huazhong University of Science and Technology, Wuhan, 430030 China; 2 Department of Forensic Medicine, Nanjing Medical University, Nanjing, 211166 China; 3 Department of Endocrinology, Union Hospital, Tongji Medical College, Huazhong University of Science and Technology, Wuhan, 430022 China; 4 Department of Pediatric Surgery, Union Hospital, Tongji Medical College, Huazhong University of Science and Technology, Wuhan, 430022 China; 5 Key Laboratory of Environment and Health (HUST), Ministry of Education, School of Public Health, Tongji Medical College, Huazhong University of Science and Technology, Wuhan, 430030 China; 6 School of Optical and Electronic Information- Wuhan National Laboratory for Optoelectronics, Huazhong University of Science and Technology, Wuhan, 430074 China; 7 College of Engineering, Huazhong Agricultural University, Wuhan, 430070 China; 8 Cancer Center, Union Hospital, Tongji Medical College, Huazhong University of Science and Technology, Wuhan, 430022 China; 9 MoE Key Laboratory for Biomedical Photonics, Huazhong University of Science and Technology, Wuhan, 430074 China

**Keywords:** PACT, CLARITY, Zebrafish, Immunofluorescence, Tyramide signal amplification, Tissue clearing, Brain imaging, Brain damage

## Abstract

For comprehensive studies of the brain structure and function, fluorescence imaging of the whole brain is essential. It requires large-scale volumetric imaging in cellular or molecular resolution, which could be quite challenging. Recent advances in tissue clearing technology (e.g. CLARITY, PACT) provide new solutions by homogenizing the refractive index of the samples to create transparency. However, it has been difficult to acquire high quality results through immunofluorescence (IF) staining on the cleared samples. To address this issue, we developed TSA-PACT, a method combining tyramide signal amplification (TSA) and PACT, to transform samples into hydrogel polymerization frameworks with covalent fluorescent biomarkers assembled. We show that TSA-PACT is able to reduce the opacity of the zebrafish brain by more than 90% with well-preserved structure. Compared to traditional method, TSA-PACT achieves approximately tenfold signal amplification and twofold improvement in signal-to-noise ratio (SNR). Moreover, both the structure and the fluorescent signal persist for at least 16 months with excellent signal retention ratio. Overall, this method improves immunofluorescence signal sensitivity, specificity and stability in the whole brain of juvenile and adult zebrafish, which is applicable for fine structural analysis, neural circuit mapping and three-dimensional cell counting.

**Supplementary Information:**

The online version contains supplementary material available at 10.1186/s13578-023-01043-1.

## Introduction

Due to the complexity of the biological systems such as the brain, it is essential to image the whole organisms or organs at cellular or molecular levels to study their structures and functions [1, 2]. It remains challenging to synchronously unite accuracy and large-scale acquirement of information through optical scanning with current technologies [3, 4]. One of the widely employed strategies is to slice the tissues and analyze layer-by-layer. However, the operation is labor-intensive with massive amount of data and inevitably leads to tissue damage, deformation, and loss of information. Especially for the brain, since the neurites extend to various directions, the neurons usually cannot be thoroughly reconstructed after section [5].

Recent advances in tissue clearing technology provide new solutions for volumetric imaging. The opacity of biological tissues stems from heterogeneous components inside tissues that mismatch the refractive index (RI) [3]. The removal of non-essential components inside tissue (clearing) contributes to homogenize RI and minimize light scattering and absorption, thereby rendering tissue transparency and accessibility to photons [6]. The mainstream tissue-clearing protocols can be classified into three technical routes: hydrophobic, hydrophilic, and hydrogel-based tissue clearing methods [3, 7–15]. Among them, the hydrogel-based tissue clearing methods (e.g. CLARITY, PACT, SHIELD, SWITCH) outstands for the stability and compatibility with bio-molecular staining [15–17]. For these methods, the intact biological tissues are substituted with hydrogel polymerization framework, where the proteins, nucleic acids and other macrobiomolecules are immobilized at their native physiological positions while the lipid components remain unbound and are able to be eluted. The decrease of lipid barrier increases the penetrability of light and antibodies, allowing the direct stereo imaging of structural and molecular phenotyping of samples without destroying the integrity [6, 13]. PACT (passive clarity technology) is a gentle hydrogel-based tissue clearing method whose degreasing speed depends on the diffusion rate of detergent micelles without extra electric field acceleration [18]. Previous studies have showed the feasibility of PACT for quickly clearing thin sections (e.g., 1–3 mm thick slices of tissues) for tissues such as the mouse brain [2, 19, 20]. For thicker tissues, the PACT procedure would take more time or requires to incorporate with a complex vasculature-based perfusion chamber [20–22].

As one of the widely used model animals, zebrafish possesses unique advantages of transparent embryos, which distinguish them out as a valuable model for studying developmental processes and modeling human diseases including neurodevelopmental disorders [23]. However, as zebrafish grows, its brain gradually becomes opaque, which obstructs the observation of the structural and molecular features inside the brain. Several attempts have been made to re-clarify zebrafish brain tissue. Since zebrafish are quite smaller than mice, PACT has been applied which enables direct detection of the fluorescent protein, and endogenous proteins through immunofluorescence [6, 24–27]. However, there is still room for improvements of these methods.

To fully record and display rich details over a broad dynamic range, strong signals and low background noise are necessary [28]. Tissue clearing methods help reduce background noises via the removal of the lipid barriers. In the meanwhile, better staining strategies still need to be developed for signal enhancement. It is worth to enhance fluorescent signal retention as it would facilitate digging up potential information through repeated imaging and long-term reanalysis [2, 29]. Moreover, multiple molecular interrogations can be performed through multiple rounds of staining and elution [6]. Herein, we introduce TSA-PACT, a method that aims to sensitively recognize multiple cellular and molecular features for whole tissues or organs. We demonstrate that TSA-PACT renders the whole brain of zebrafish transparent, compatible with bio-molecular staining. Furthermore, compare to existing strategies, TSA-PACT improves signal amplification, SNR, long-term signal retention and enables multiple rounds of labeling in volumetric imaging. With TSA-PACT, we achieved high-resolution, high-content mapping of neuron morphology and distribution for juvenile and adult zebrafish brain. Therefore, this method could contribute to the exploration of native components and functional relationships of the central nervous system.

## Method

### Experimental animals

Zebrafish (Tu strain) were purchased from the Institute of Hydrobiology, Chinese Academy of Sciences and kept in the recirculation aquaculture systems in standardized raising conditions (28 ℃, 14 h light/10 h dark cycle) for 30–40 days (juvenile) or 3–4 months (adult). For the chronic hyperglycemia (CHG) brain damage model, zebrafish embryos were raised for 14 days and then placed in fish water supplemented with 111 mM D-glucose for another 14 days [30]. The glucose solution was renewed every day to prevent the deterioration of water quality. All experiments were approved by the Medical Ethics Committee at Tongji Medical College, Huazhong University of Science and Technology.

### Sample preparation

Zebrafish were anesthetized in the 100 mg/L MS-222 (tricaine methane sulfonate) solution, followed by decapitation operation after zebrafish became unconscious [31]. Brain tissues were dissected out and fixed in the paraformaldehyde (PFA) solution (4% PFA in PBS) at 4 ℃ for at least 1 day.

### PACT clearing for brain tissue of zebrafish

Fixed brain tissues were incubated in the A4P0 hydrogel solution (4% acrylamide in PBS) supplemented with 0.25% thermal initiator 2,2′-azobis [2-(2-imidazolin-2-yl)propane] dihydrochloride (VA-044, MACKLIN, A824730) at 4 ℃ for 1–2 day. Before polymerization, A4P0-incubated samples underwent nitrogen deaeration operation for 3–5 min. Samples were incubated in a thermostat water bath at 37 ℃ to initiate tissue-hydrogel hybridization, lasting for 3–4 h. These hydrogel-transformed tissues were placed into the clearing buffer (8% SDS in 0.2 M Boric Acid Buffer at pH 8.5) at 37 ℃ with gentle shaking for passive lipid removal. It took approximately 5–10 days until the entire tissue became transparent. The tissues were then washed for 3 times with PBST (PBS containing 0.2% Tween) at room temperature (RT) over the course of one day to wash away the SDS residue.

### Immunofluorescence staining of PACT-processed brain tissue of zebrafish

Clarified brain tissues were used to perform immunofluorescence staining. Samples were incubated in the blocking buffer [PBST containing 2% normal goat serum, 2% bovine serum albumin and 0.1% proclin-300 (Genemill, GB01003S)] at RT overnight. Samples were transferred to the blocking buffer containing primary antibody (Additional file 4: Table S1) and incubated at 37 ℃ with gentle shaking in a thermostat shaker for 5–7 days, followed by 3 time PBST washing over the course of one day. Samples were incubated in the blocking buffer containing secondary antibody (Additional file 4: Table S2) at 37 ℃ with gentle shaking for 5–7 days, followed by 3 time PBST washing over the course of one day to remove the unbound antibodies.

### Tyramide signal amplification of HRP-labeled brain tissue of zebrafish

HRP-labeled tissues were transferred to Tris Buffer (1 M, pH 7.4) and immersed by TSA reaction solution (Alexa Fluor^™^ 647 tyramide reagent (1:100, Invitrogen^™^, B40958), Cyanine 3 tyramide reagent (1:100, Perkinelmer, NEL744001KT) or Fluorescein tyramide reagent (1:100, Perkinelmer, NEL741001KT) in Tris Buffer) at RT overnight. 0.01% H_2_O_2_ was added to initiate the catalytic reaction. The reaction lasted for 1–2 h. Tissues were washed 3 times with PBST to stop the reaction and to remove the unbound TSA reagent.

### Multiple labeling using TSA-PACT

To perform TSA-PACT with two or more antibodies, the samples post staining were washed with the clearing buffer for one day to denature and remove the resident antibodies. The samples were then washed 3 times with PBST at RT to wash away the SDS residue. Samples were then incubated with another primary antibody and stained with another tyramide reagent. All operations were performed in the absence of light.

### Refractive index homogenization

85% glycerol was utilized as a refractive index matching solution (RIMS) to homogenize the RI of tissue [10, 18]. Tissues were immersed in the RIMS overnight for imaging and were able to be stored at 4 ℃ for several months.

### Imaging and data analysis

To measure the structural integrity and transparency, samples were immersed in 4% PFA (pre-clearing) or RIMS (post-clearing), and imaged with ZEISS AXIO Zoom.V16 microscope with black grids as background. The size of the samples was outlined using ZEN (v3.2). Images were converted to 8-bit format, inverted, and the gray value of the grid was measured using ImageJ. Transparency was determined by calculating the ratio of the averaged gray value of the grids in five regions under the samples to that in five no-sample-regions.

Fluorescence images were obtained with ZEISS LSM 800 confocal microscope using a 10X objective or 20X objective at 488 nm (FITC), 561 nm (Cy3) or 640 nm (Alexa Fluor^™^ 647) excitation. Tissues were immersed in RIMS, using confocal dishes as containers. For comparisons between IF-PACT and TSA-PACT, images were acquired at an interval of 5 μm within 300 μm tissue depth. The threshold function of ImageJ software was used to distinguish the signal from background, and the signal-to-noise ratio was determined by calculating the ratio of mean fluorescence intensity of the signal to the mean intensity of the rest of the image. Normalized mean fluorescence quantification was calculated by the ratio of the sum of the pixel intensity to the sum of pixel number in the signal minus that of the background, to demonstrate the quality of the signal over the background [32, 33]. To quantify fluorescence level in three-dimension, max projections were conducted for every 50 μm, followed by SNR analysis. For tiled images, images were imported into ImageJ to perform stitching of tile scans via the stitching plugin. The 3D digital reconstruction was conducted by the Imaris software. The Spots module of Imaris was used to automatically count cells within certain regions of the brain.

### Statistical statement

Data were present as mean ± standard error of the mean (S.E.M). Statistical tests were performed using the SPSS software. Paired *t* test was used to evaluate the size of samples before and after clearing. Unpaired *t* test was used to compare clarification ability between glycerol and PACT, fluorescence intensity and normalized mean fluorescence between IF-PACT and TSA-PACT, and the number of pHH3-positive cells between CHG zebrafish and controls. One-way ANOVA test was used to compare SNR in concentration gradients experiment, followed by Bonferroni post hoc test.

## Results

### Tissue clearing of zebrafish brain

**Fig. 1:**
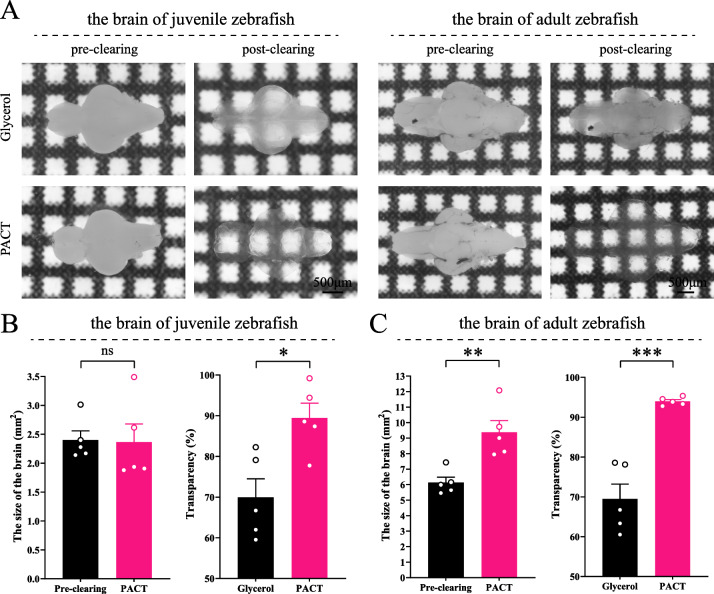
Clarified zebrafish brain achieves optimal transparency and structural integrity. **A** Images of the brain of juvenile and adult zebrafish pre-clearing and post-clearing (85% glycerol or PACT). **B** and **C** Size changes after PACT clearing and the difference of transparency between glycerol clearing and PACT clearing in juvenile zebrafish brain (**B**) and adult zebrafish brain (**C**). Statistical significance in size changes and transparency difference was assessed by paired *t* test and unpaired *t* test, respectively. (mean ± S.E.M.; n = 5; ns, *p* > 0.05; *p < 0.05; **p < 0.01; and ***p < 0.001)

The brain of zebrafish appears to be milky and opaque since juvenile. The size of the brain is approximately 1 mm × 1 mm × 2.5 mm in juvenile zebrafish and 1.5 mm × 1.5 mm × 4 mm in adult zebrafish, which are suitable for the PACT clearing strategies (Fig. 1A). We tested several hydrogel incubation protocols and found that 4% PFA fixation followed by 4% acrylamide incubation protocol (A4P0) is optimal for zebrafish brains [18]. We also tried to use 85% glycerol as clearing reagent, however the resulted tissue transparency was significantly lower (Fig. 1B, C). We modified the original protocol by increasing the volume of clearing buffer (1 mL/brain). With higher volume, it is not necessary to change the buffer throughout the clearing process, which simplified the operation. To further enhance the transparency, we tested different recipes of RIMS and selected 85% glycerol, which is cost-effective, easy to prepare, of low toxicity and compatible with various fluorescent staining procedures. With the modified protocol, we were able to reduce the opacity by 90–94% for the zebrafish brain. Besides, the structural integrity of samples was well preserved post clarification, without either excessive swelling or shrinkage (Fig. 1B and C). The detailed procedure is provided in the Additional file 3.

### Combining TSA with PACT improves staining quality

**Fig. 2:**
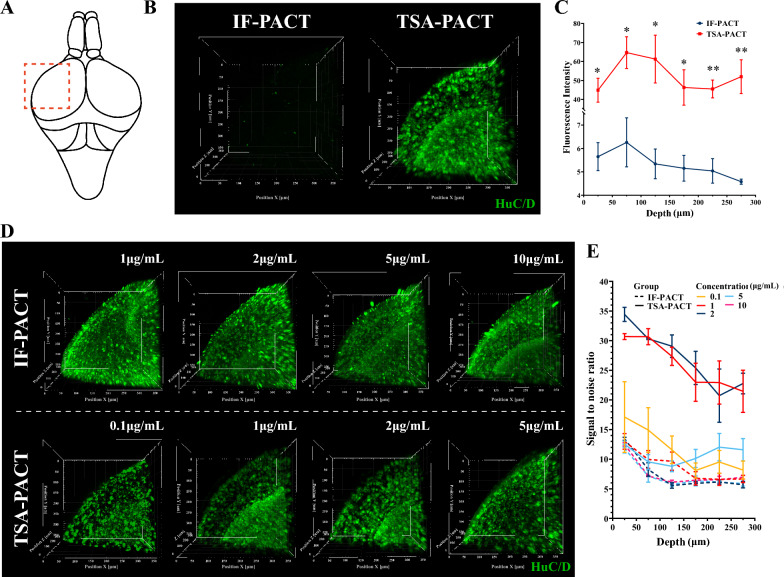
TSA-PACT allows signal amplification and SNR optimization. **A** A sketch of the structure of zebrafish brain. The box indicates the optic tectum, the imaged area for (**B**) and (**D**). **B** and **C** Three-dimensional images (**B**) and fluorescence intensity of the signals in different depth (**C**) in optic tectum with HuC/D staining post-IF-PACT/TSA-PACT. For different clearing methods, the same staining and imaging condition were used for the same regions. Samples were all incubated in 1 μg/mL HuC/D antibody. Images were acquired by 5 μm interval with the same microscope parameters (0.1% wave length; 100 μm pinhole; 600 V detector gain). Fluorescence intensity of signals was calculated from each 50 μm-z projection. The level of significance was calculated by unpaired Student’s t-test. (Voxel size: 0.624 μm × 0.624 μm × 5 μm; mean ± S.E.M.; n = 3; *p < 0.05; **p < 0.01). **D** and **E** Three-dimensional images (**D**) and signal-to-noise ratio in different depth (**E**) in optic tectum with HuC/D staining post-IF-PACT/TSA-PACT. Samples were incubated in the HuC/D antibody of 0.1, 1, 2, 5 or 10 μg/mL. Images were captured by 5 μm interval within the 300 μm depth, using the optimized parameters of confocal microscope (100 μm pinhole; 600 V detector gain; 1 μg/mL IF-PACT, 4% wave length; 2 μg/mL IF-PACT, 4% wave length; 5 μg/mL IF-PACT, 3% wave length; 10 μg/mL IF-PACT, 2% wave length; 0.1 μg/mL TSA-PACT, 0.7% wave length; 1 μg/mL TSA-PACT, 0.1% wave length; 2 μg/mL TSA-PACT, 0.1% wave length; 5 μg/mL TSA-PACT, 0.1% wave length). Signal-to-noise ratio was calculated from each 50 μm-z projection. The level of significance was calculated by one-way ANOVA test, followed by Bonferroni post hoc test, shown in Additional file 4: Table S3. (Voxel size: 0.624 μm × 0.624 μm × 5 μm; mean ± S.E.M.; n = 3)

Post clarification, it is critical to gather imaging data at high resolution and high SNR to obtain high-detailed information at cellular or molecular levels of brain-wide perspective. To improve SNR, previous PACT strategies have been focused on noise elimination rather than signal enhancement. Therefore, we applied TSA to immunostaining post PACT (TSA-PACT) aiming to improve signal intensity and SNR. To examine the effect of TSA-PACT on signal enhancement, we compared the imaging results using traditional IF staining method (IF-PACT) or TSA-PACT in side by side experimental setting. For HuC/D staining in the optic tectum, the fluorescence intensity using TSA-PACT was 8–13 fold to that using IF-PACT with the same antibody concentrations, incubation time, and confocal settings (Fig. 2A–C).

To determine if TSA-PACT could improve SNR, we performed IF-PACT and TSA-PACT with gradient concentrations of HuC/D and GFAP antibody and optimized confocal microscopy setting for each preparation to obtain the best SNR of each method (Fig. 2D, Additional file 1: Figure S1A). The results showed that TSA-PACT outperformed IF-PACT to achieve high SNR (Fig. 2E, Additional file 1: Figure S1B). The best SNR using the TSA-PACT method was more than two-fold of that using IF-PACT method. Moreover, compared with IF-PACT, TSA-PACT required lower antibody concentration to produce high-quality images (100μL 1:2000-diluted GFAP antibody solution is able to label up to five brains in TSA-PACT). These data showed that TSA-PACT is able to amplify target signals much more than nonspecific signals. Importantly, unnecessarily high concentration of primary antibody (5 µg/mL for HuC/D, 1:100 dilution for GFAP) is not recommended in TSA-PACT, probably due to the high sensitivity of the method which greatly increases the noise caused by nonspecific binding or leftover of the excessive antibodies. Taken together, the TSA-PACT method significantly improves the sensitivity and specificity of immunostaining in zebrafish brains post clarification.

### Long-term storage of post staining samples using TSA-PACT

**Fig. 3:**
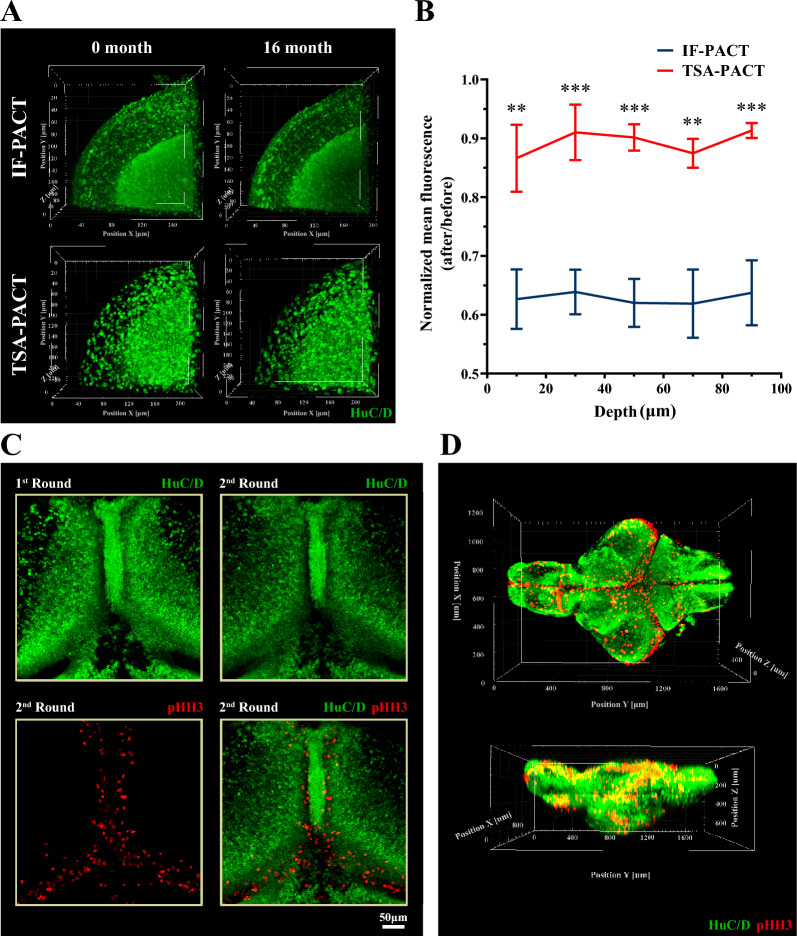
TSA-PACT allows long-term signal retention and multiple molecular interrogations. **A** and **B** Three-dimensional images (**A**) and normalized mean fluorescence in different depth (**B**) in optic tectum with HuC/D staining taken at 0 and 16 months post IF-PACT/TSA-PACT. IF-PACT samples were incubated in 5 μg/mL HuC/D antibody and acquired images with 1% wave length, 100 μm pinhole and 650 V detector gain. TSA-PACT samples were incubated in 2 μg/mL HuC/D antibody and acquired images with 0.2% wave length; 41 μm pinhole; 560 V detector gain. Samples were held in 85% glycerol at 4 °C. After 16 months, they were re-analyzed using the previous microscope parameter. The level of significance was calculated by unpaired Student’s *t*-test. (Voxel size: 0.312 μm × 0.312 μm × 2 μm; mean ± S.E.M.; n = 5; **p < 0.01; and ***p < 0.001). **C** Images of multiple-round staining with anti-HuC/D antibody (green; first round) and anti-pHH3 antibody (red; second round). **D** Three-dimensional view of the whole brain with HuC/D and pHH3 staining in juvenile zebrafish. (Voxel size: 1.25 μm × 1.25 μm × 5 μm; green, HuC/D; red, pHH3)

Next, we examined whether TSA-PACT enables long-term storage of samples post immunofluorescence staining, which is critical for repeated imaging and long-term reanalysis of the tissue. Theoretically, TSA-PACT has advantages over IF-PACT in signal retention, because tyramide is covalently assembled to the hydrogel-tissue hybridization system whereas the fluorescence conjugated secondary antibodies rely on the noncovalent intermolecular forces of antigen–antibody binding. We immersed samples into 85% glycerol and re-detected their fluorescence signals after 16 months. Indeed, samples processed by TSA-PACT outperformed in the maintenance of normalized mean fluorescence compare to those by IF-PACT after 16 months (Fig. 3A and B). These results demonstrated the persistence of fluorescence signals with TSA-PACT method.

### Multi-molecular staining through multiple rounds of labeling

Frequently, it requires to simultaneously label two or more biomolecules for structural or functional studies of the brain. Therefore, we developed a multiple labelling strategy. Since tyramide is covalently binding to hydrogel–tissue hybridization network whereas the antibodies are noncovalently bond to the antigen, the delipidation treatment would probably remove the antibodies but not the tyramide. Therefore, after the first round of staining, we incubated the samples with 8% SDS clearing solution to denature and remove the primary and secondary antibodies and then performed the second round of staining. As the proof-of-principle, we double labelled the zebrafish brain with HuC/D and pHH3 antibodies (Fig. 3C). The result showed that after harsh elution treatment, the first round HuC/D fluorescence signals remained in situ with approximately 9–17% quenching. Meanwhile, the native antigenicity of tissues was not disrupted, allowing subsequent pHH3 staining. Besides, there was no nonspecial co-staining in the secondary staining, indicating that the residual antibodies in the first-round staining were thoroughly removed. An integrated map of the morphology and distribution of HuC/D and pHH3 positive neurons was generated throughout the intact brain (Fig. 3D and Additional file 2: Movie S1).

### Application of TSA-PACT to examine brain structures in physiological and pathological conditions

**Fig. 4:**
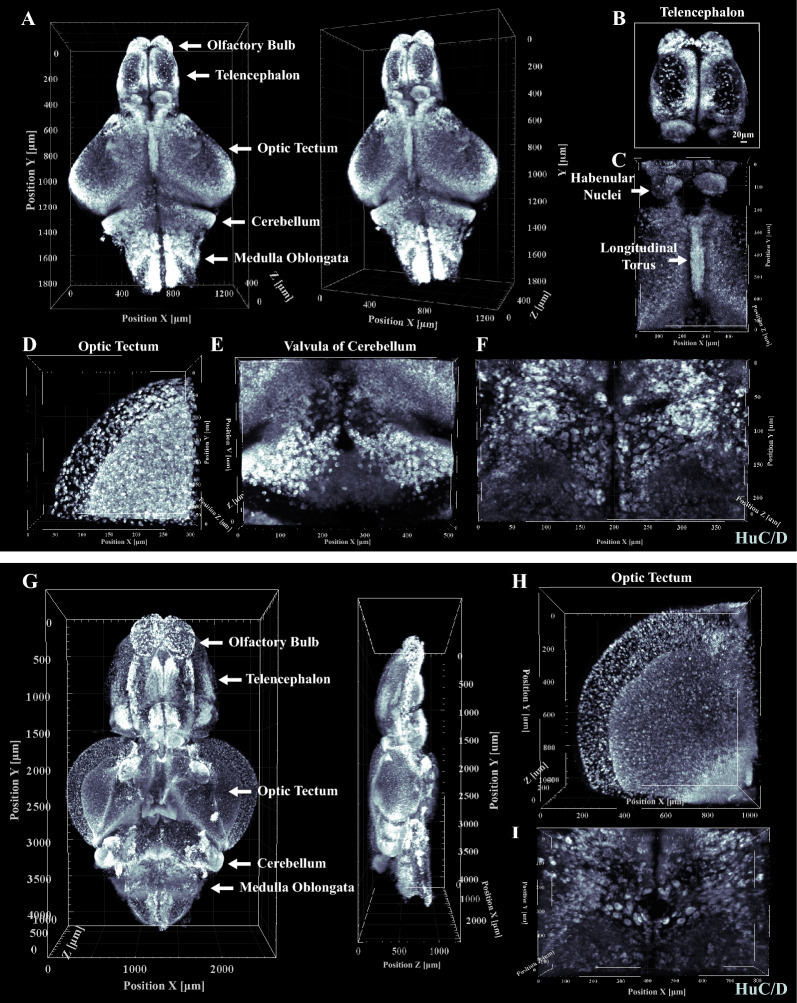
Zebrafish brain structural/cellular phenotyping with HuC/D staining. **A** Three-dimensional view of the whole brain with HuC/D staining in juvenile zebrafish. Several brain regions can be dissected, including olfactory bulb, telencephalon, optic tectum, cerebellum and medulla oblongata. (Voxel size: 1.25 μm × 1.25 μm × 5 μm). **B**–**F** Images of telencephalon, habenular nuclei and longitudinal torus, optic tectum, valvula of cerebellum with HuC/D staining in juvenile zebrafish. Neurons with large cell bodies were accumulated under the longitudinal torus. (Voxel size: 1.25 μm × 1.25 μm × 1 μm for **C** and **E**; Voxel size: 0.312 μm × 0.312 μm × 1 μm for **D**; Voxel size: 0.624 μm × 0.624 μm × 1 μm for **F**). **G** Three-dimensional view of the whole brain with HuC/D staining in adult zebrafish. Several brain regions can be recognized, including olfactory bulb, telencephalon, optic tectum, cerebellum and medulla oblongata. (Voxel size: 1.25 μm × 1.25 μm × 5 μm). **H** and **I** Images of optic tectum and neurons with large cell bodies with HuC/D staining in adult zebrafish. (Voxel size: 1.25 μm × 1.25 μm × 5 μm)

We then tried to analyze the brain structure in different conditions. In wild type brain with HuC/D staining, we were able to identify several regions of the juvenile and adult zebrafish brain, such as olfactory bulb, telencephalon, optic tectum, diencephalon, cerebellum and medulla oblongata (Fig. 4A and G, Additional file 2: Movie S4). In the telencephalon, HuC/D positive cells were scattered but denser in the midline (Fig. 4B). A pair of symmetric habenular nuclei could be discerned between telencephalon and optic tectum in dorsal view (Fig. 4C). In the optic tectum, the positive cells became increasingly dense from the surface to the inner layer (Fig. 4D and H, Additional file 2: Movie S2). Below the optic tectum, neurons with large cell bodies were symmetrically accumulated along the midline of diencephalon (Fig. 4F and I, Additional file 2: Movie S3). A longitudinal torus was located between the bilateral optic tectums (Fig. 4C). Below the longitudinal torus, a specialized structural valvula of cerebellum could be recognized as continuous with the cerebellum (Fig. 4E). The 3D assembly of HuC/D labeled neurons enabled structural and cellular analysis of the brain with both high resolution and whole-tissue perspective.

**Fig. 5:**
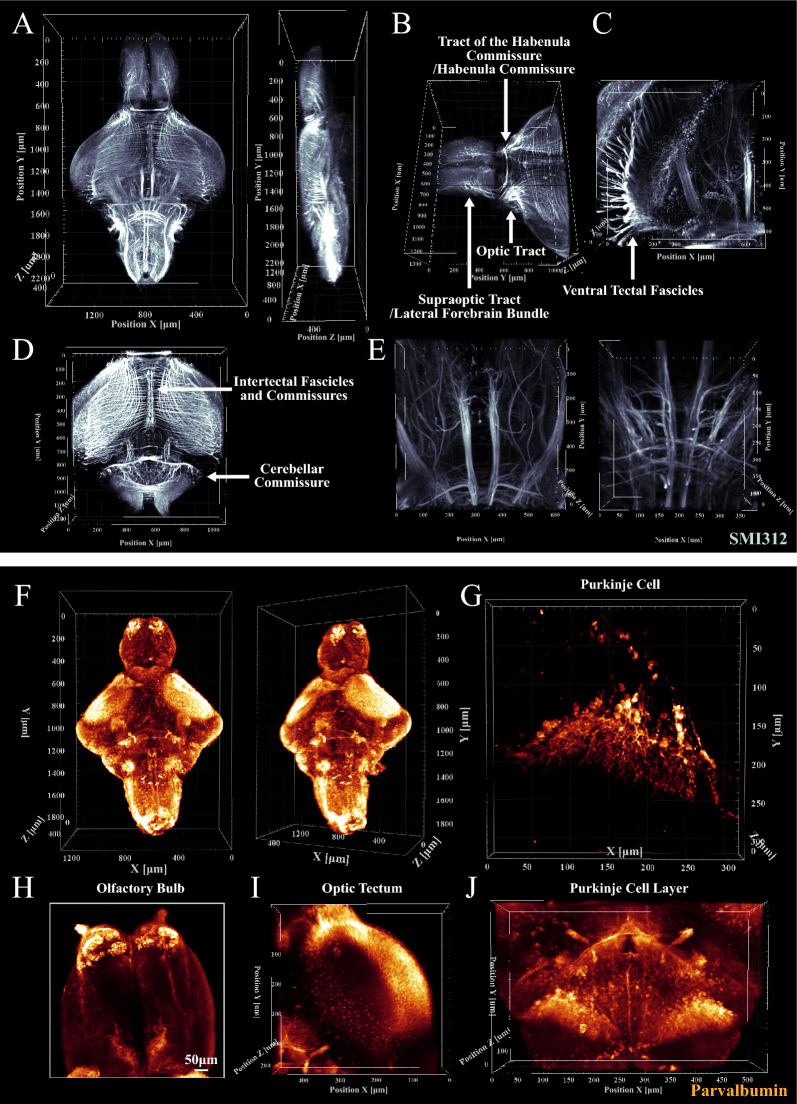
Zebrafish brain structural/cellular phenotyping with SMI312 and PV staining. **A** Three-dimensional view of the whole brain with SMI312 staining in juvenile zebrafish. The structure and distribution of major neurofilaments were displayed of brain-wide perspective. (Voxel size: 1.25 μm × 1.25 μm × 2 μm). **B**–**E** Images of supraoptic tract/lateral forebrain bundle, tract of the habenula commissure/habenula commissure, optic tract, ventral tectal fascicles, intertectal fascicles and commissures, cerebellar commissure and the ascending and descending pathway in juvenile zebrafish. (Voxel size: 1.25 μm × 1.25 μm × 2 μm for **B** and **D**; Voxel size: 0.624 μm × 0.624 μm × 2 μm for **C** and **E**). **F** Three-dimensional view of the whole brain with PV staining in juvenile zebrafish. (Voxel size: 1.25 μm × 1.25 μm × 5 μm). **G**–**J** Images of Purkinje cells, Purkinje cell layer and axonal tracts in the cerebellum, and PV-positive cells in olfactory bulb and optic tectum. Purkinje cells are with a big pear-shaped cell body and dendritic trees. They were gathered in Purkinje cell layer. (Voxel size: 0.312 μm × 0.312 μm × 1 μm for **G**; Voxel size: 1.25 μm × 1.25 μm × 2 μm for **I** and **J**)

To characterize the neuronal morphology, we labeled intermediate filaments with SMI312 antibody. The structure and distribution of major neurofilaments were clear in brain-wide perspective (Fig. 5A and Additional file 2: Movie S5) [34, 35]. Axonal tracts showed a symmetrical distribution (Fig. 5A). Supraoptic tract/lateral forebrain bundle were located at the lateral of telencephalon. A thick tract of the habenular commissure stretched across the left and right symmetrical habenular nuclei. Optic tracts passed through the anterior midbrain and terminated in the optic tectum (Fig. 5B) [36]. Claw-shaped ventral tectal fascicles were also connected to the optic tectum (Fig. 5C). Intertectal fascicles and commissures and cerebellar commissure interconnected directly the two sides of the optic tectum and cerebellar hemispheres, respectively (Fig. 5D). Hindbrain is an important junction for the ascending and descending pathway, where axonal tracts showed a complex crisscross pattern (Fig. 5E).

Next, we performed parvalbumin (PV) immunostaining to visualize the morphology and distribution of Purkinje cells in the cerebellar cortex and successfully identified PV-positive neurons (Fig. 5F). We observed highly specialized Purkinje cells with a big pear-shaped cell body and dendritic trees (Fig. 5G). They were gathered to form a Purkinje cell layer in the cerebellum (Fig. 5J and Additional file 2: Movie S6). The axonal tracts were also highlighted in the cerebellum, which provided the groundwork for identifying neuronal circuitries in the cerebellum (Fig. 5J). Except for the cerebellum, PV-positive staining was also present in the olfactory bulb and optic tectum (Fig. 5H and I).

**Fig. 6:**
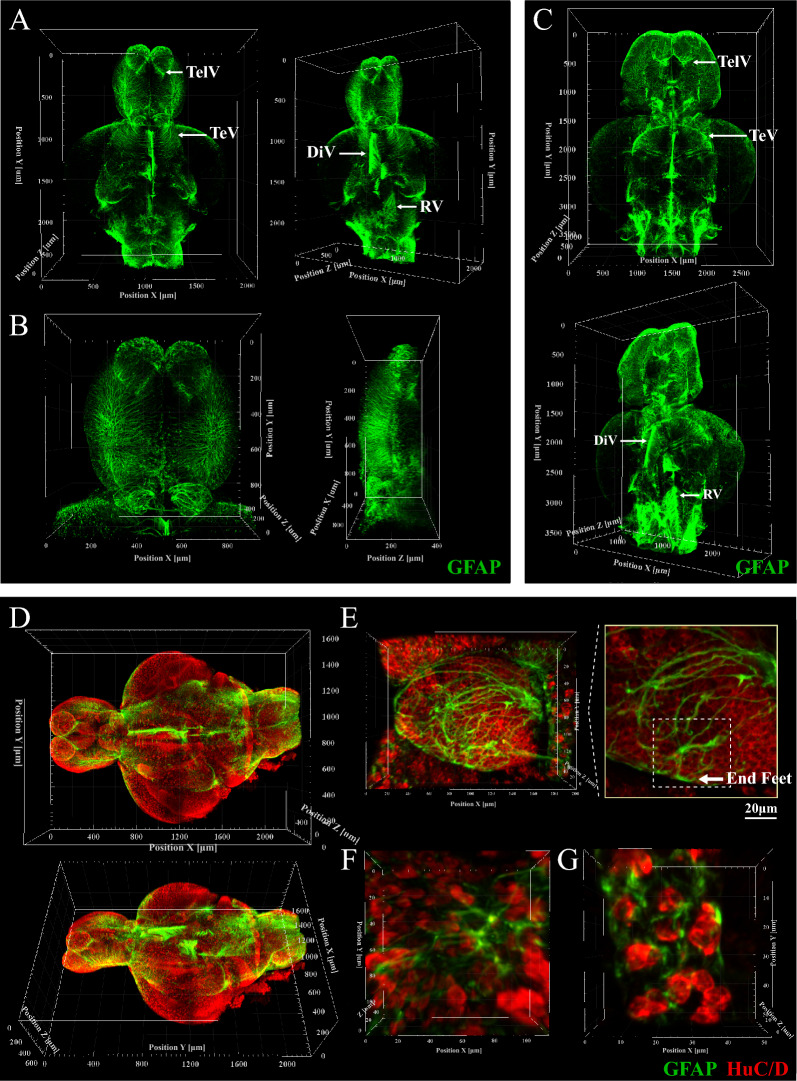
Zebrafish brain structural/cellular phenotyping with GFAP staining. **A** and **B** Three-dimensional view of the whole brain (**A**) and telencephalon (**B**) with GFAP staining in juvenile zebrafish. The shape and precise location of astrocytes in the CNS were displayed in brain-wide perspective. (Voxel size: 1.25 μm × 1.25 μm × 3 μm for A; Voxel size: 0.624 μm × 0.624 μm × 3 μm for B). **C** Three-dimensional view of the whole brain with GFAP staining in adult zebrafish. The shape and precise location of astrocytes in the CNS were displayed in brain-wide perspective. (Voxel size: 1.25 μm × 1.25 μm × 5 μm). **D** Three-dimensional view of the whole brain with GFAP and HuC/D staining in juvenile zebrafish. GFAP-positive cells are located in most regions of the brain, running radially from the ventricle to the pial surface (Voxel size: 1.25 μm × 1.25 μm × 3 μm; green, GFAP; red, HuC/D). **E**–**G** Images of neuron and astrocytes with HuC/D and GFAP staining in juvenile zebrafish. Astrocytes showed long processes radiating from the cell body and the terminal expansions of cytoplasmic processes called end feet. Astrocytes are located among neurons and interact with several neighbor neurons. (Voxel size: 0.312 μm × 0.312 μm × 2 μm for **E** and **F**; Voxel size: 0.078 μm × 0.078 μm × 2 μm for G; green, GFAP; red, HuC/D)

We also performed GFAP staining, which labels a unique intermediate filaments protein in astrocytes, in both juvenile zebrafish and adult zebrafish to outline the shape and precise location of astrocytes in the CNS (Fig. 6A and C, Additional file 2: Movies S7, S8). GFAP-positive cells showed some long processes radiating from the central cell body (Fig. 6B and E, Additional file 2: Movie S10). Some cells exhibited terminal expansions of cytoplasmic processes, termed as end feet (Fig. 6E). GFAP-positive cells are located in most regions of the brain, running radially from the ventricle to the pial surface, especially in the telencephalic ventricles (TelV), diencephalic ventricle (DiV), tectal ventricle (TeV) and rhombencephalic ventricle (RV) (Fig. 6A and C) [37, 38]. To demonstrate the relationship between neurons and astrocytes, we performed GFAP and HuC/D double staining (Fig. 6D and Additional file 2: Movie S9). Astrocytes could be observed to be located among neurons and to interact with several neighbor neurons (Fig. 6F and G).

**Fig. 7:**
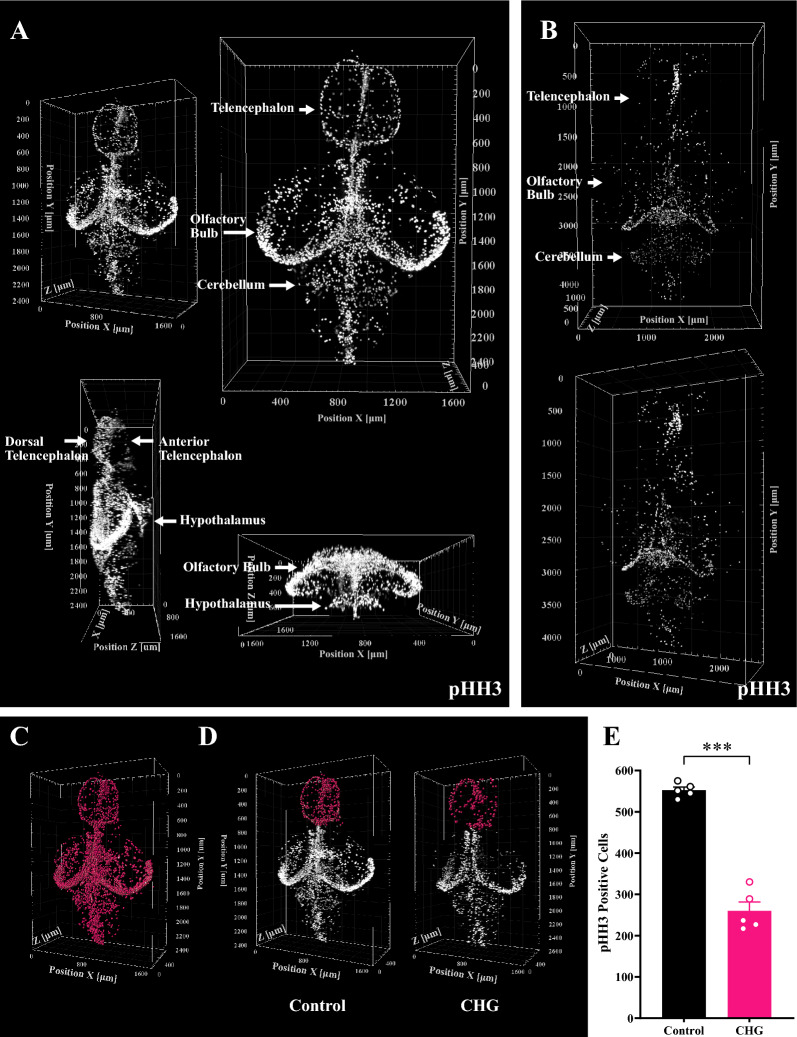
Alterations of zebrafish brain proliferation pattern in a chronic hyperglycemia brain damage model. **A** Three-dimensional view of the whole brain with pHH3 staining of juvenile zebrafish. (Voxel size: 1.25 μm × 1.25 μm × 5 μm). **B** Three-dimensional view of the whole brain with pHH3 staining of adult zebrafish. (Voxel size: 1.25 μm × 1.25 μm × 5 μm). **C** Three-dimensional pHH3-positive cell counting analysis throughout the brain. The pHH3-positive cells were automatically identified and marked with pink spot. (Voxel size: 1.25 μm × 1.25 μm × 5 μm). **D** Three-dimensional pHH3-positive cell counting analysis for the telencephalon in chronic hyperglycemia (CHG) zebrafish and controls. CHG zebrafish were kept in fish water supplemented with 111 mM D-glucose over a period of 14 days. The pHH3-positive cells were automatically identified and marked with pink spot. (Voxel size: 1.25 μm × 1.25 μm × 5 μm). **E** Comparison of pHH3-positive cells in the telencephalon between CHG zebrafish and controls. The level of significance is calculated by unpaired Student’s *t*-test (mean ± S.E.M.; n = 5; ***p < 0.001)

The proliferation pattern reflects the development and plasticity of the brain at various life stages, therefore we labeled the pHH3–positive cells to depict the overall distribution of proliferating cells in the brain of juvenile and adult zebrafish (Fig. 7A and B, Additional file 2: Movie S11). In wild type juvenile brain, these cells spread over the surface of the dorsal telencephalon and gathered at the anterior portion of the ventral telencephalon. In the mesencephalon, they were concentrated on the edge of tectum opticum. These cells are densely existed in the thalamus and hypothalamus whereas scattered throughout the cerebellum. In wild type adult brain, pHH3-positive cells were less distributed on the surface and were mostly located along the rostro-caudal axis, especially in the ventricular area [39, 40]. In the telencephalon, there is a prominent proliferation zone located along midline. In the mesencephalon, the proliferation zone extended around the margin of the optic tectum and toward posterior part of TeV. Whereas in the cerebellum, proliferating cells are scattered. Moreover, TSA-PACT staining enables three-dimensional cell counting throughout the brain (Fig. 7C). We generated a chronic hyperglycemia injury model by keeping the fish in high glucose for 14 days, which could induce metabolic disorders, affecting brain inflammation and neurogenesis [30, 41]. We then performed cell counting in the telencephalon, which is sensitive to chronic hyperglycemia (Fig. 7D). High glucose treated zebrafish displayed significantly reduction of pHH3–positive cells by 50% compared with controls (Fig. 7E). The results showed that the TSA-PACT method could be used to generate high quality data for brain-related studies.

## Discussion

**Fig. 8:**
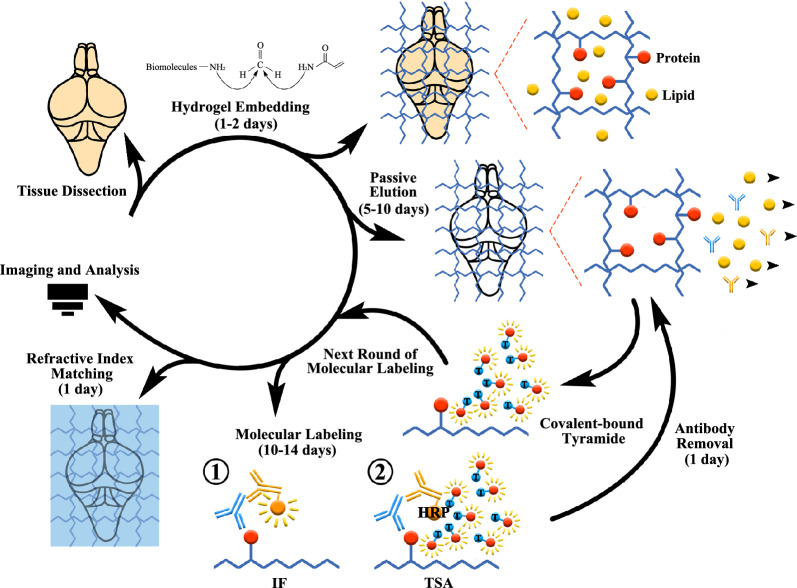
Workflow of TSA-PACT. The brains are dissected out, embedded in hydrogel, eluted passively, fluorescence labeled for one or multiple rounds, matched refractive index and finally imaged using confocal, light sheet microscope, or other 3D imaging platforms

Recently, much effort has been made to enable the imaging of intact tissues/organs to answer important biological questions. The tissue clearing technique emerged as a strategy for direct volumetric imaging via optical refractive index matching. Various tissue clearing techniques and their derivatives have been developed, aiming to improve imaging sensitivity, specificity and long-term signal retention. In this study, we introduced TSA-PACT, a method aiming to improve the quality of immunofluorescence staining for post clearing tissues, which has been a bottleneck for tissue clearing procedures (Fig. 8). We showed that TSA-PACT rendered the whole zebrafish brain transparent, compatible with bio-molecular staining, and outperformed the traditional IF-PACT method in signal amplification, SNR enhancement, long-term signal retention and multiple molecule labeling. We demonstrated the advantages of TSA-PACT with high-resolution, high-content brain imaging. We also applied it into redraw brain structure, neural networks and the alterations of proliferation pattern in a disease model.

TSA-PACT is originated from PACT, a gentle hydrogel-based tissue clearing method substituting passive diffusion for electrophoresis. Electrophoresis might cause epitope denaturation, fine structure damage and tissue browning due to the electrical heat. In contrast, the gentle degreasing procedure in PACT could help to control these damages to a minimum [18]. Although the clearing speed is slower, PACT is suitable for the zebrafish brain with reasonable clearing time.

For penetration of antibodies into clarified brain samples, the diffusion time depends on the sample volume [3]. For zebrafish brain, the staining procedure could be implemented by passive diffusion. For subjects of bigger size, the incubation time needs to be significantly prolonged or rapid staining strategies based on stochastic electro transport for homogeneous labeling have to be applied [42]. Alternatively, nanobodies could be used, which are single-domain antibody fragments with a molecular weight of only 12–15 kDa [43]. The small size allowed them to freely make way through the pores of the hydrogel, promoting fast tissue permeability [20].

To identify the alternations and abnormalities of the brain structure in various pathological conditions, it is essential to draw an accurate brain map [44]. It requires acquirement of as many independent subjects as possible to assemble a reference brain and its neuroanatomical segmentation [45]. TSA-PACT permits the mass storage and long-term re-analysis of samples for its stability of both structure and fluorescent signal. Moreover, its capability of multi-molecular labeling fosters a more detailed neuroanatomical segmentation and molecular position. These features offer a powerful tool for brain studies.

## Supplementary Information

**Additional file 1: Figure S1.** TSA-PACT allows SNR optimization with GFAP staining. Three-dimensional imagesand signal-to-noise ratio in different depthin telencephalon with GFAP staining post IF-PACT or TSA-PACT. Samples were incubated in the GFAP antibody of 1:100, 1:500 or 1:2000 dilution. Images were captured by 2 μm interval, using the optimized parameters of confocal microscope. Signal-to-noise ratio was calculated from each 50 μm-z projection. The level of significance was calculated by one-way ANOVA test, followed by Bonferroni post hoc test, shown in **Additional file 4: Table S4.**

**Additional file 2: Movie S1.** Animation of the intact brain with HuC/Dand pHH3staining in juvenile zebrafish. Movie S2. Animation of optic tectum with HuC/D staining in juvenile zebrafish. Movie S3. Animation of HuC/D-positive neurons with large cell bodies in juvenile zebrafish. Movie S4. Animation of the intact brain with HuC/D staining in adult zebrafish. Movie S5. Animation of the intact brain with SMI312 staining in juvenile zebrafish. Movie S6. Animation of cerebellum with PV staining in juvenile zebrafish. Movie S7. Animation of the intact brain with GFAP staining in juvenile zebrafish. Movie S8. Animation of the intact brain with GFAP staining in adult zebrafish. Movie S9. Animation of the intact brain with HuC/Dand GFAPstaining in juvenile zebrafish. Movie S10. Animation of neuron and astrocytes with HuC/Dand GFAPstaining in juvenile zebrafish. Movie S11. Animation of the intact brain with pHH3 staining in juvenile zebrafish.

**Additional file 3:** TSA-PACT Protocol.

**Additional file 4: Table S1.** Primary antibodies used in this study. **Table S2**. Secondary antibodies used in this study.** Table S3.** Statistical significance of SNR in different depth in optic tectum with anti-HuC/D staining by IF-PACT or TSA-PACT. **Table S4.** Statistical significance of SNR in different depth in telencephalon with GFAP staining by IF-PACT or TSA-PACT.

## Data Availability

All analyzed data supporting the findings of this study are available within the article. The raw data including confocal images are available from the corresponding authors, H.S. and B.X., upon reasonable request.

## Abbreviations

IF: Immunofluorescence
TSA: Tyramide signal amplification
SNR: Signal-to-noise ratio
RI: Refractive index
RT: Room temperature
RIMS: Refractive index matching solution
PV: Parvalbumin
pHH3: Phospho-histone H3
TelV: Telencephalic ventricles
DiV: Diencephalic ventricle
TeV: Tectal ventricle
RV: Rhombencephalic ventricle
