# Supplementary figures and images for: TSA-PACT: a method for tissue clearing and immunofluorescence staining on zebrafish brain with improved sensitivity, specificity and stability
Source: Cell Biosci. 2023 May 26;13:97. doi: 10.1186/s13578-023-01043-1 (PMC10223841; doi:10.1186/s13578-023-01043-1)

**A****IF-PACT****1:100 dilution**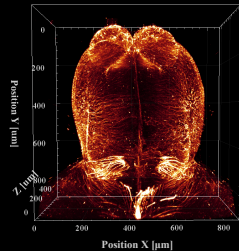**1:500 dilution**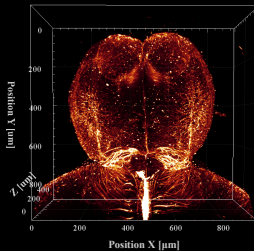**1:2000 dilution**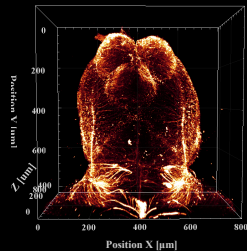**1:100 dilution**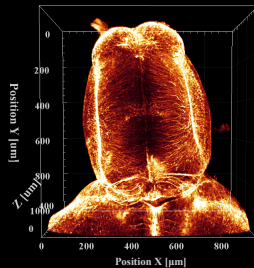**1:500 dilution**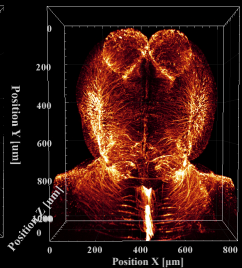**1:2000 dilution**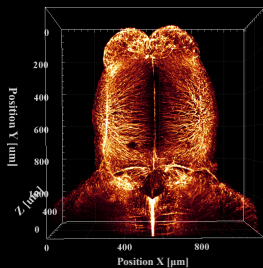**TSA-PACT****B**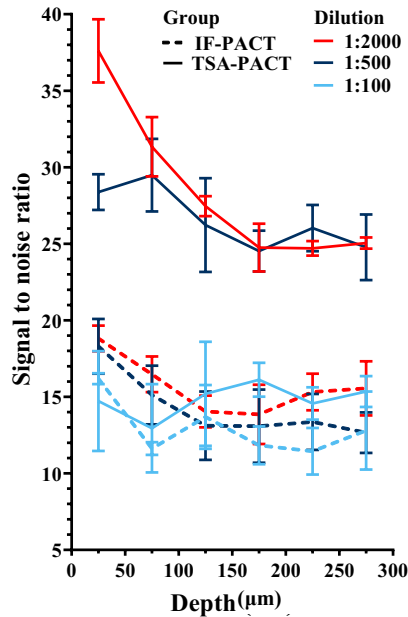

Supplement: Supplementary file 1 — Additional file 1: Figure S1. TSA-PACT allows SNR optimization with GFAP staining. Three-dimensional imagesand signal-to-noise ratio in different depthin telencephalon with GFAP staining post IF-PACT or TSA-PACT. Samples were incubated in the GFAP antibody of 1:100, 1:500 or 1:2000 dilution. Images were captured by 2 μm interval, using the optimized parameters of confocal microscope. Signal-to-noise ratio was calculated from each 50 μm-z projection. The level of significance was calculated by one-way ANOVA test, followed by Bonferroni post hoc test, shown in Additional file 4: Table S4. [file 13578_2023_1043_MOESM1_ESM.pdf]
