# Supplementary material for: TSA-PACT: a method for tissue clearing and immunofluorescence staining on zebrafish brain with improved sensitivity, specificity and stability
Source: Cell Biosci. 2023 May 26;13:97. doi: 10.1186/s13578-023-01043-1 (PMC10223841; doi:10.1186/s13578-023-01043-1)
Supplement: Supplementary file 3 — Additional file 3: TSA-PACT Protocol. [file 13578_2023_1043_MOESM3_ESM.docx]

**TSA-PACT Protocol**

1. Anesthetize zebrafish in 100 mg/L MS-222 (tricaine methane sulfonate) solution, followed by decapitation operation after zebrafish became unconscious.
2. Isolate the brain under microscope and fix it in 4% PFA at 4℃ for at least 1 day.
3. Incubate the samples in 10 ml hydrogel solution at 4℃ for 1 day (juvenile zebrafish) or 2 days (adult zebrafish).

*Note*:

- 1. Keep all reagents on ice during the whole procedure.
  2. Acrylamide monomer is toxic. Wear rubber gloves while performing all operations.
  3. Tweezer is easy to damage and contaminate samples. We recommend plastic dropper instead of tweezers to transfer samples.

**Hydrogel solution**

| Chemical | Mass in 10 mL | Final concentration |
| --- | --- | --- |
| Acrylamide | 0.4 g | 4% |
| VA-044 | 0.025 g | 0.25% |
| PBS (1x) | To 10 mL | 1x |

1. Perform the degas operation in a 15 mL centrifuge tube sealed with parafilm. Connect a MPPI-3 microinjection pump with a nitrogen tank and a thin tube. Open the main valve of nitrogen tank and adjust its control valve to produce a stable and slow nitrogen flow. Put the thin tube into liquid after piercing the parafilm. Fill the hydrogel solution with nitrogen for 3-5 minutes to eliminate dissolved oxygen. After pumping sufficient nitrogen, cap the tube quickly.

*Note*: Avoid the splashing of toxic hydrogel solution caused by too strong nitrogen flow.


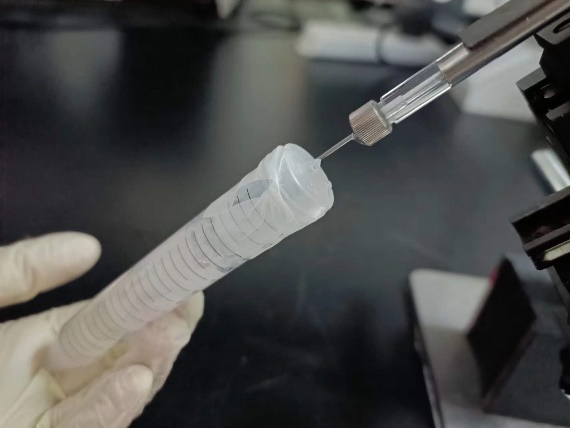


1. Incubate the samples in a 37℃-water-bath for 3-4 hours.

*Note*: A4P0 transforms tissue into a stable hydrogel–tissue hybridization form without hydrogel outside tissue. It avoids the laborious operation of separating redundant hydrogel outside tissue.

1. Transfer the samples into 15-50 mL clearing buffer. Gently shake the sample at 37 °C to remove lipid. It usually takes 5-10 days until the entire tissue became transparent.

*Note*: It is not necessary to replace the clearing buffer for zebrafish brain during the delipidation process.

**Clearing buffer (pH = 8.5)**

| Chemical | Mass in 500 mL | Final concentration |
| --- | --- | --- |
| Boric acid buffer (5x) | 100 mL | 1x |
| SDS | 40 g | 8% |
| H_2_O | To 500 mL |  |

**Boric acid buffer (5x)**

| Chemical | Mass in 500 mL |
| --- | --- |
| Na_2_B_4_O_7_•10H_2_O | 21.45 g |
| Borate | 17.0 g |
| H_2_O | To 500 mL |

1. Wash the samples with PBST (PBS containing 0.2% Tween) for 3 times (2-3 hours each time) at room temperature (RT).

*Note*: SDS in the clearing buffer should be completely removed, or it will affect antibody binding.

1. Incubate the samples in the blocking solution at RT overnight.

*Note*: Proclin-300 can efficiently inhibit the growth of bacteria, which is essential to protect samples from spoiling. It will be better to use 0.22 µm filter membrane to remove bacteria in the blocking solution.

**Blocking solution**

| Chemical | Mass in 5 mL | Final concentration |
| --- | --- | --- |
| Normal goat serum | 100 μL | 2% |
| Bovine serum albumin | 0.1 g | 2% |
| Proclin-300 | 5 μL | 0.1% |
| PBST | To 5 mL |  |

1. Incubate the samples in primary antibody/blocking solution (Table S1) at 37℃ with gentle shaking for 5-7 days.

***Note*: We recommend 2 mL Eppendorf tube to incubate tissues, where 100-200 μL antibody/blocking solution is enough for molecular labeling.**

**List of antibodies tested in PACT tissue:**

**http://wiki.claritytechniques.org/index.php/Immunostaining#Primary_antibodies_used_in_CLARITY_literature**

1. Wash away the primary antibody with PBST for 3 times (2-3 hours each time) at RT.
2. Incubate the samples in secondary antibody/blocking solution (Table S2) at 37℃ with gentle shaking for 5-7 days.

***Note*: We recommend 2 mL Eppendorf tube to incubate tissues, where 100-200 μL antibody/blocking solution is enough for molecular labeling.**

1. Wash away the secondary antibody with PBST for 3 times (2-3 hours each time) at RT.
2. Transfer the samples to Tris Buffer (1M, pH 7.4).
3. Immerse the samples with TSA reaction solution (Dilute tyramide 1:100 in Tris Buffer) overnight. ***(only for TSA-PACT)***
4. Add 0.01% H_2_O_2_ to TSA reaction solution for 1.5 hours at 20℃. ***(only for TSA-PACT)***

*Note*:

- 1. High temperature will over-accelerate the catalytic reaction, leading to too strong outer signal. It is not conductive to dyeing evenness.
  2. We recommend 2 mL Eppendorf tube to incubate tissues, where 50 μL TSA reaction solution is enough for catalytic reaction.

1. Wash away the TSA reagent with PBST for 3 times (30 min each time) at RT. ***(only for TSA-PACT)***
2. To perform another round of labeling, wash the samples post staining in clearing buffer at 37℃ with gentle shaking for 1 days. Repeat step 7, 9-16 for multiple labeling. ***(Optional)***
3. Incubate the samples in 85% glycerol overnight.

*Note*: The samples can be stored in 85% glycerol at 4 °C for at least 1 year.

1. Scan the samples on imaging platforms.
