# Supplementary material for: TSA-PACT: a method for tissue clearing and immunofluorescence staining on zebrafish brain with improved sensitivity, specificity and stability
Source: Cell Biosci. 2023 May 26;13:97. doi: 10.1186/s13578-023-01043-1 (PMC10223841; doi:10.1186/s13578-023-01043-1)
Supplement: Supplementary file 4 — Additional file 4: Table S1. Primary antibodies used in this study. Table S2. Secondary antibodies used in this study. Table S3. Statistical significance of SNR in different depth in optic tectum with anti-HuC/D staining by IF-PACT or TSA-PACT. Table S4. Statistical significance of SNR in different depth in telencephalon with GFAP staining by IF-PACT or TSA-PACT. [file 13578_2023_1043_MOESM4_ESM.docx]

| **Table S1. Primary antibodies used in this study** | | | |
| --- | --- | --- | --- |
| Antibody | Host Species | Supplier | Recommended Dilution |
| GFAP | Mouse | Sigma-Aldrich, G3893 | 1:100 (IF-PACT)  1:2000 (TSA-PACT) |
| HuC/HuD | Mouse | Invitrogen, A-21271 | 5-10 μg/mL (IF-PACT)  1-2 μg/mL (TSA-PACT) |
| Neurofilament (SMI312) | Mouse | Biolegend, 837904 | 1:2000 (TSA-PACT) |
| Phospho-Histone H3 (Ser10) | Rabbit | Cell Signal Technology, 9701 | 1:2000 (TSA-PACT) |
| Parvalbumin | Rabbit | GeneTex, GTX134110 | 1:500 (TSA-PACT) |

| **Table S2. Secondary antibodies used in this study** | | | |
| --- | --- | --- | --- |
| Antibody | Host Species | Supplier | Recommended Dilution |
| Alexa Fluor™ Plus 647, anti-Mouse IgG (H+L) Antibody | Mouse | Invitrogen, A32728 | 1:200 (IF-PACT) |
| HRP conjugated Rabbit Anti-Goat IgG (H+L) | Rabbit | Servicebio, GB23303 | 1:200 (TSA-PACT) |
| HRP conjugated Mouse Anti-Goat IgG (H+L) | Mouse | Servicebio, GB23301 | 1:200 (TSA-PACT) |

| **Table S3. Statistical significance of SNR in different depth in optic tectum with anti-HuC/D staining by IF-PACT or TSA-PACT** | | | | | | | | | | | |
| --- | --- | --- | --- | --- | --- | --- | --- | --- | --- | --- | --- |
| Depth  (μm) | Group | Concentration  (μg/mL) | IF-PACT  (μg/mL) | | | |  | TSA-PACT  (μg/mL) | | | |
|  |  |  | 1 | 2 | 5 | 10 |  | 0.1 | 1 | 2 | 5 |
| 0-50 | IF-PACT | 1 |  | 1 | 1 | 1 |  | 1 | 0.001 | <0.001 | 1 |
|  |  | 2 |  |  | 1 | 1 |  | 1 | 0.001 | <0.001 | 1 |
|  |  | 5 |  |  |  | 1 |  | 1 | 0.001 | <0.001 | 1 |
|  |  | 10 |  |  |  |  |  | 1 | 0.001 | <0.001 | 1 |
|  | TSA-PACT | 0.1 |  |  |  |  |  |  | 0.015 | 0.001 | 1 |
|  |  | 1 |  |  |  |  |  |  |  | 1 | 0.001 |
|  |  | 2 |  |  |  |  |  |  |  |  | <0.001 |
| 0 |  | 5 |  |  |  |  |  |  |  |  |  |
| 50-100 | IF-PACT | 1 |  | 1 | 1 | 1 |  | 1 | <0.001 | <0.001 | 1 |
|  |  | 2 |  |  | 1 | 1 |  | 0.338 | <0.001 | <0.001 | 1 |
|  |  | 5 |  |  |  | 1 |  | 0.126 | <0.001 | <0.001 | 1 |
|  |  | 10 |  |  |  |  |  | 0.128 | <0.001 | <0.001 | 1 |
|  | TSA-PACT | 0.1 |  |  |  |  |  |  | <0.001 | <0.001 | 1 |
|  |  | 1 |  |  |  |  |  |  |  | 1 | <0.001 |
|  |  | 2 |  |  |  |  |  |  |  |  | <0.001 |
|  |  | 5 |  |  |  |  |  |  |  |  |  |
| 100-150 | IF-PACT | 1 |  | 1 | 1 | 1 |  | 1 | <0.001 | <0.001 | 1 |
|  |  | 2 |  |  | 1 | 1 |  | 0.191 | <0.001 | <0.001 | 1 |
|  |  | 5 |  |  |  | 1 |  | 0.234 | <0.001 | <0.001 | 1 |
|  |  | 10 |  |  |  |  |  | 0.344 | <0.001 | <0.001 | 1 |
|  | TSA-PACT | 0.1 |  |  |  |  |  |  | <0.001 | <0.001 | 1 |
|  |  | 1 |  |  |  |  |  |  |  | 1 | <0.001 |
|  |  | 2 |  |  |  |  |  |  |  |  | <0.001 |
|  |  | 5 |  |  |  |  |  |  |  |  |  |
| 150-200 | IF-PACT | 1 |  | 1 | 1 | 1 |  | 1 | <0.001 | <0.001 | 1 |
|  |  | 2 |  |  | 1 | 1 |  | 1 | <0.001 | <0.001 | 1 |
|  |  | 5 |  |  |  | 1 |  | 1 | <0.001 | <0.001 | 1 |
|  |  | 10 |  |  |  |  |  | 1 | <0.001 | <0.001 | 1 |
|  | TSA-PACT | 0.1 |  |  |  |  |  |  | 0.001 | <0.001 | 1 |
|  |  | 1 |  |  |  |  |  |  |  | 1 | 0.002 |
|  |  | 2 |  |  |  |  |  |  |  |  | <0.001 |
|  |  | 5 |  |  |  |  |  |  |  |  |  |
| 200-250 | IF-PACT | 1 |  | 1 | 1 | 1 |  | 1 | 0.004 | 0.017 | 1 |
|  |  | 2 |  |  | 1 | 1 |  | 1 | 0.003 | 0.013 | 1 |
|  |  | 5 |  |  |  | 1 |  | 1 | 0.003 | 0.013 | 1 |
|  |  | 10 |  |  |  |  |  | 1 | 0.004 | 0.016 | 1 |
|  | TSA-PACT | 0.1 |  |  |  |  |  |  | 0.027 | 0.109 | 1 |
|  |  | 1 |  |  |  |  |  |  |  | 1 | 0.131 |
|  |  | 2 |  |  |  |  |  |  |  |  | 0.524 |
|  |  | 5 |  |  |  |  |  |  |  |  |  |
| 250-300 | IF-PACT | 1 |  | 1 | 1 | 1 |  | 1 | <0.001 | <0.001 | 1 |
|  |  | 2 |  |  | 1 | 1 |  | 1 | <0.001 | <0.001 | 0.677 |
|  |  | 5 |  |  |  | 1 |  | 1 | <0.001 | <0.001 | 0.804 |
|  |  | 10 |  |  |  |  |  | 1 | <0.001 | <0.001 | 1 |
|  | TSA-PACT | 0.1 |  |  |  |  |  |  | 0.001 | <0.001 | 1 |
|  |  | 1 |  |  |  |  |  |  |  | 1 | 0.021 |
|  |  | 2 |  |  |  |  |  |  |  |  | 0.007 |
|  |  | 5 |  |  |  |  |  |  |  |  |  |

| **Table S4. Statistical significance of SNR in different depth in telencephalon with GFAP staining by IF-PACT or TSA-PACT** | | | | | | | | | | |
| --- | --- | --- | --- | --- | --- | --- | --- | --- | --- | --- |
| Depth  (μm) | Group | Dilution | IF-PACT | | |  | TSA-PACT | | |  |
|  |  |  | 1:100 | 1:500 | 1:2000 |  | 1:100 | 1:500 | 1:2000 |  |
| 0-50 | IF-PACT | 1:100 |  | 1 | 1 |  | 1 | 0.008 | <0.001 |  |
|  |  | 1:500 |  |  | 1 |  | 1 | 0.033 | <0.001 |  |
|  |  | 1:2000 |  |  |  |  | 1 | 0.048 | <0.001 |  |
|  | TSA-PACT | 1:100 |  |  |  |  |  | 0.003 | <0.001 |  |
|  |  | 1:500 |  |  |  |  |  |  | 0.061 |  |
|  |  | 1:2000 |  |  |  |  |  |  |  |  |
| 50-100 | IF-PACT | 1:100 |  | 1 | 1 |  | 1 | <0.001 | <0.001 |  |
|  |  | 1:500 |  |  | 1 |  | 1 | 0.003 | 0.001 |  |
|  |  | 1:2000 |  |  |  |  | 1 | 0.008 | 0.002 |  |
|  | TSA-PACT | 1:100 |  |  |  |  |  | 0.001 | <0.001 |  |
|  |  | 1:500 |  |  |  |  |  |  | 1 |  |
|  |  | 1:2000 |  |  |  |  |  |  |  |  |
| 100-150 | IF-PACT | 1:100 |  | 1 | 1 |  | 1 | 0.035 | 0.018 |  |
|  |  | 1:500 |  |  | 1 |  | 1 | 0.025 | 0.013 |  |
|  |  | 1:2000 |  |  |  |  | 1 | 0.042 | 0.021 |  |
|  | TSA-PACT | 1:100 |  |  |  |  |  | 0.081 | 0.04 |  |
|  |  | 1:500 |  |  |  |  |  |  | 1 |  |
|  |  | 1:2000 |  |  |  |  |  |  |  |  |
| 150-200 | IF-PACT | 1:100 |  | 1 | 1 |  | 1 | 0.002 | 0.002 |  |
|  |  | 1:500 |  |  | 1 |  | 1 | 0.006 | 0.005 |  |
|  |  | 1:2000 |  |  |  |  | 1 | 0.01 | 0.008 |  |
|  | TSA-PACT | 1:100 |  |  |  |  |  | 0.056 | 0.046 |  |
|  |  | 1:500 |  |  |  |  |  |  | 1 |  |
|  |  | 1:2000 |  |  |  |  |  |  |  |  |
| 200-250 | IF-PACT | 1:100 |  | 1 | 0.952 |  | 1 | <0.001 | <0.001 |  |
|  |  | 1:500 |  |  | 1 |  | 1 | <0.001 | 0.001 |  |
|  |  | 1:2000 |  |  |  |  | 1 | 0.002 | 0.005 |  |
|  | TSA-PACT | 1:100 |  |  |  |  |  | 0.001 | 0.003 |  |
|  |  | 1:500 |  |  |  |  |  |  | 1 |  |
|  |  | 1:2000 |  |  |  |  |  |  |  |  |
| 250-300 | IF-PACT | 1:100 |  | 1 | 1 |  | 1 | 0.005 | 0.004 |  |
|  |  | 1:500 |  |  | 1 |  | 1 | 0.004 | 0.003 |  |
|  |  | 1:2000 |  |  |  |  | 1 | 0.035 | 0.028 |  |
|  | TSA-PACT | 1:100 |  |  |  |  |  | 0.03 | 0.024 |  |
|  |  | 1:500 |  |  |  |  |  |  | 1 |  |
|  |  | 1:2000 |  |  |  |  |  |  |  |  |
